# Electronic Modulation of THz Radiation at NovoFEL: Technical Aspects and Possible Applications

**DOI:** 10.3390/ma12193063

**Published:** 2019-09-20

**Authors:** Oleg A. Shevchenko, Anatoly R. Melnikov, Sergey V. Tararyshkin, Yaroslav V. Getmanov, Stanislav S. Serednyakov, Evgeny V. Bykov, Vitaly V. Kubarev, Matvey V. Fedin, Sergey L. Veber

**Affiliations:** 1 Budker Institute of Nuclear Physics of the Siberian Branch of the Russian Academy of Sciences, 630090 Novosibirsk, Russia; 2 International Tomography Center of the Siberian Branch of the Russian Academy of Sciences, 630090 Novosibirsk, Russia; 3 Voevodsky Institute of Chemical Kinetics and Combustion of the Siberian Branch of the Russian Academy of Sciences, 630090 Novosibirsk, Russia; 4 Novosibirsk State University, 630090 Novosibirsk, Russia

**Keywords:** free-electron laser, NovoFEL, THz radiation, electronic modulation, macropulse, EPR spectroscopy

## Abstract

The Novosibirsk Free Electron Laser (NovoFEL) facility is able to produce high-power tunable terahertz (THz) laser radiation in quasi-continuous mode. The ability to control/shape this THz radiation is required in a number of user experiments. In this work we propose a modulation approach suitable for free electron lasers based on recuperation design. It allows for generating THz macropulses of a desirable length, down to several microseconds (limited by a quality factor of FEL optical resonator). Using this approach, macropulses in the time window from several microseconds to several hundred microseconds have been shown for three possible frequency ranges: mid-infrared (~1100 cm^−1^), far-infrared (~200 cm^−1^) and THz (~40 cm^−1^). In each case, the observed rise and decay of the macropulse have been measured and interpreted. The advantage of using short macropulses at the maximum peak power available has been demonstrated with the time-resolved Electron Paramagnetic Resonance (EPR) spectroscopy.

## 1. Introduction

The Novosibirsk Free Electron Laser (NovoFEL) facility comprises three FELs, which use an energy recovery linac (ERL) as a source of electrons. It operates in quasi-continuous (CW) mode and produces a periodic train of radiation pulses. Three FELs of the NovoFEL facility are designed to generate the laser light in THz (1st FEL), far-infrared (2nd FEL), and mid-infrared (3rd FEL) ranges (for uniformity, all of them are referred to as THz radiation) [1]. Energy recovery allows one to achieve a very high average current of the electron beam, which results in high average power of radiation. Although this is crucial for some applications [2], in most of the experiments, such high average power is excessive and thus should be attenuated. In particular cases, the use of short macropulses is required which allows one to keep the peak power very high, while the average one can be reduced significantly [3]. To fulfill these requirements without the use of external devices (e.g., mechanical optical choppers), one should be able to switch on and off the FEL lasing process. For the case of FELs based on linear accelerators and thus operating in pulsed mode [4], the FEL lasing can be controlled by the electron beam injection process (which is normally working in pulse mode). When FEL is optimized for lasing quasi-continuous radiation, the switching on and off of the electron beam injection is not a suitable way to control the power of THz radiation, since an ERL is not developed for such a regime. Particularly, for the case of the NovoFEL accelerator, beam loading effects in the accelerating structure are very significant because of the high average current: fast switching of the current leads to transient processes resulting in beam loss. Instead of that, a more “delicate” approach should be developed that keeps the electron beam current almost constant, but efficiently suppresses/enhances the THz generation process. In this work, we developed and implemented at NovoFEL the approach that allows for generating the THz macropulses at any repetition rate and almost any individual length from several seconds down to several microseconds (limited by a quality factor of the FEL optical resonator). This regime has been examined at all three FELs by generation of macropulses with tens of microseconds duration. Possible applications of the electronic modulation of THz radiation are discussed with particular consideration of time-resolved EPR spectroscopy. The latter was used to trace the spin dynamics of copper(II) complex reflecting the sample temperature changes on a microsecond timescale.

## 2. Materials and Methods

**Figure 1:**
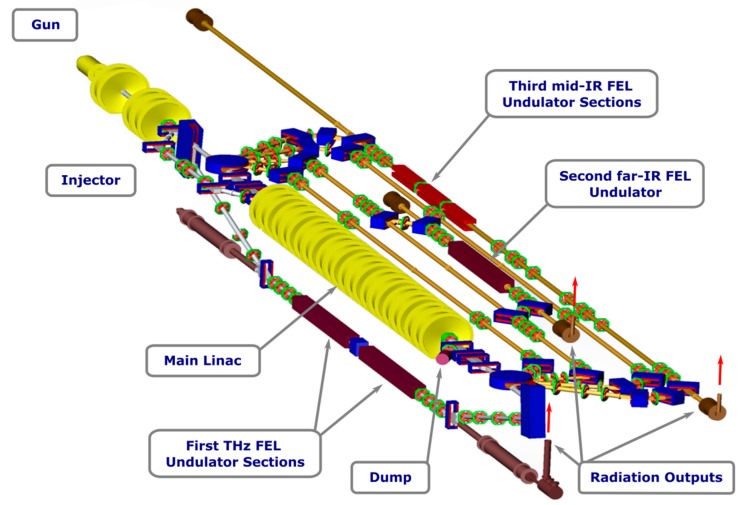
Main assemblies of the Novosibirsk Free Electron Laser (NovoFEL) facility and their spatial layout.

A general view of the NovoFEL facility is shown in Figure 1. It includes injector, main linac and beamlines where FEL undulators are installed. There is one beamline in the vertical plane and four beamlines in the horizontal plane. The first FEL uses the vertical beamline. The second and the third FEL undulators are installed on the second and the fourth horizontal beamlines, respectively. The detailed description of the facility can be found elsewhere [1].

**Figure 2:**
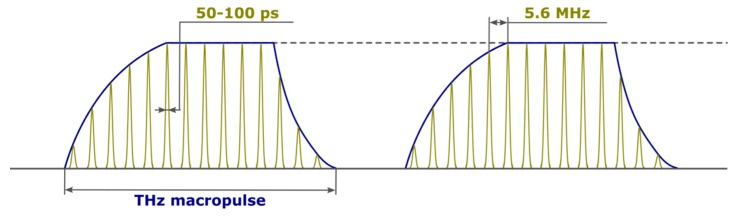
Schematic view of NovoFEL radiation macropulses. The length of the individual THz pulse and pulse repetition rate are shown for 1st FEL.

All three FELs of the NovoFEL facility are FEL-oscillators. A typical example of the time-dependence of the first FEL radiation is shown in Figure 2. In the normal operation regime (CW), this structure is a continuous train of short radiation pulses (50–100 ps), which follow each other with the frequency of ~5.6 MHz determined by the optical cavity length. When NovoFEL operates in the power modulation mode, the laser radiation consists of macropulses with minimal duration of about 10 µs and arbitrary repetition rate (user-controlled). Each macropulse contains tens of individual THz radiation pulses and its fronts depend on the FEL gain (pulse rise) and the quality factor of the optical cavity (pulse decay).

**Figure 3:**
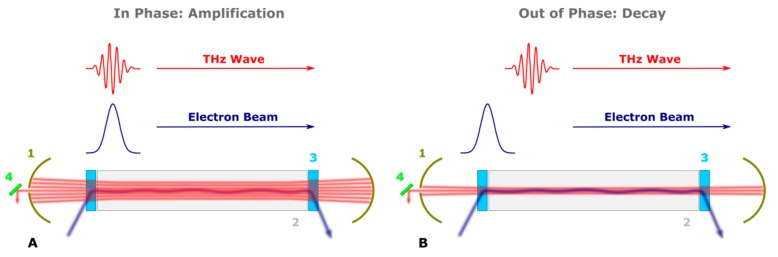
Schematic representation of THz radiation amplification (**A**) or suppression (**B**). The latter is achieved by periodic shift of the phase of the electron bunch injection. The numbers show optical resonator (1), undulator (2), dipole magnets (3) and THz radiation output (4).

In the normal operation regime (CW mode), THz radiation and electron bunches in the optical resonator are required to come to the undulator simultaneously (Figure 3A). In that case, stimulated emission occurs, allowing for the amplification of THz radiation. Thus, THz light amplification in the FEL oscillator takes place only when (i) the electron bunch repetition rate is equal to the round-trip frequency of the optical cavity and (ii) the electron bunch and optical wave have “in phase” propagation in the optical cavity (Figure 3A). When NovoFEL operates in the power modulation mode, THz light amplification should be suppressed in the time between THz macropulses, and this can be done by the suppression of the stimulated emission process. Thus, THz light suppression in the FEL oscillator can be achieved when (i) the electron bunch repetition rate is still equal to the round-trip frequency of the optical cavity, but (ii) the electron bunch is “phase-shifted” to the optical wave in the optical cavity (Figure 3B). The latter is achieved when the injection phase of the electron bunch is shifted by, for example, one period of the radio frequency RF accelerating field, which almost does not influence on beam dynamics in the accelerator. To prevent the formation of a new THz wave, this injection phase shift has to be done periodically. The corresponding change of the bunch repetition rate required for suppression is less than 1% and does not lead to any noticeable beam loading effects.

**Figure 4:**
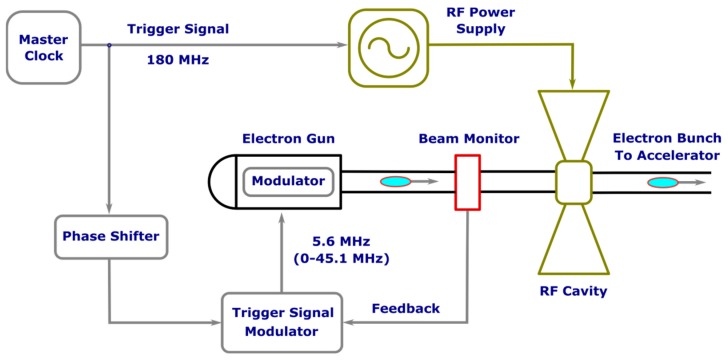
The injection phase shifting system.

The scheme of practical realization of the injection phase shifting is shown in Figure 4. Injection is triggered by the signal of the modulator, which counts the pulses coming from the master clock. Pulses come with the repetition rate equal to RF accelerating field frequency 180.4 MHz. The simplest algorithm to operate the trigger signal of the modulator is the following: it cuts N-1 successive pulses out of the 180.4 MHz pulse sequence and then triggers injection with the next Nth pulse (for the first FEL N = 32, for the second FEL N = 24 and for the third FEL N = 48). In a normal regime (CW), the phase shifter is switched off, while in power modulation mode it is on. The phase shifter operation algorithm is the following: it periodically cuts each Kth pulse out of the 180.4 MHz pulse sequence, which results in the shift of the injection phase by one RF period. The smaller the values of K, the better suppression of FEL lasing is achieved. At the same time, K should be large enough to avoid influence on accelerator operation. It was shown experimentally that K = 100 is sufficient to suppress FEL lasing completely without influencing the accelerator operation.

Time-resolved electron paramagnetic resonance (TR EPR) experiments under THz radiation were performed on an X-band (~9 GHz) EPR spectrometer based on commercial microwave (MW) bridge ER 046MRPTW and ER 4118X-MD5 resonator (both Bruker, Karlsruhe, Germany). The setup is located in the user hall of NovoFEL (Figure S1 in Supporting Information). The standard dielectric sapphire insert in the resonator was replaced by a bismuth germanate one with 4 mm inner diameter [5]. Low temperature experiments were performed using helium cryostat (Cryotrade Engineering, Moscow, Russia) and LakeShore 335 temperature controller (Lake Shore Cryotronics, Westerville, OH, USA). MW frequency was controlled by Agilent 53131A-124 frequency counter (Agilent, Santa Clara, CA, USA). Time-resolved EPR signals were recorded by LeCroy 9350AM oscilloscope (Teledyne Technologies, Thousand Oaks, CA, USA) connected to a PC by the fsc2 program [6]. THz radiation of samples inside the resonator was carried out via a specially designed waveguide, which was also used as a sample holder. The average power of THz radiation in TR EPR experiments was measured by a Gentec-EO UP19K-15S-VR detector (Gentec-EO, Quebec, QC, Canada). TR EPR experiments were carried out using an electronic modulation system, unless otherwise stated. A more detailed description of the experimental setup can be found elsewhere [3].

Electronic modulation of THz radiation was triggered by an arbitrary waveform generator (AWG) of Keysight DSOX3034T oscilloscope (Keysight Technologies, Santa Rosa, CA, USA). The rectangular pulses from the oscilloscope AWG with an arbitrary chosen repetition rate (typically 3–20 Hz) were synchronized with the electron beam frequency by a home-build D-trigger scheme. The electron beam frequency was measured in real time by pick-up coil. The synchronization with pick-up coil signal allows us to detect the fine structure of THz macropulses and resolve individual pulses of NovoFEL radiation when recording the waveform in averaging mode. The same pick-up-synchronized signal from AWG was used for triggering LeCroy oscilloscope to start TR EPR measurement. All characterization of THz macropulses and TR EPR experiments were done using the pick-up-synchronized signal, unless otherwise stated.

Experimental setup used for visualization of the THz macropulses can be schematically described as trigger signal (AWG + D-trigger) → FEL → THz detector → oscilloscope. As a THz detector, we used the Schottky-barrier detector (first FEL) [7], Scontel superconducting bolometer type 1a (Scontel, Moscow Russia) (second FEL), and MCT LN_2_ mid-IR detector (InfraRed Associates, FL USA) (third FEL). At the first and third FELs, Keysight DSOX3034T (Keysight Technologies, Santa Rosa, CA, USA) were used as an oscilloscope, while LeCroy Wavemaster 830Zi (Teledyne Technologies, Thousand Oak, CA, USA) was used at the second FEL. Spectra of radiation used at the first and second FELs were measured using a modernized MDR-23 monochromator (Lomo Photonica, St. Petersburg, Russia) and MG-33 pirosensor (Vostok, Moscow, Russia) installed on a support that is moved horizontally by a stepper motor controlled by PC [8]. At the third FEL, radiation spectrum was measured by fourier-transform infrared spectrometer Vertex 70v (Bruker Optics, Leipzig, Germany). The spectra are shown in Figures S2, S5 and S8 of the Supporting Information.

**Figure 5:**
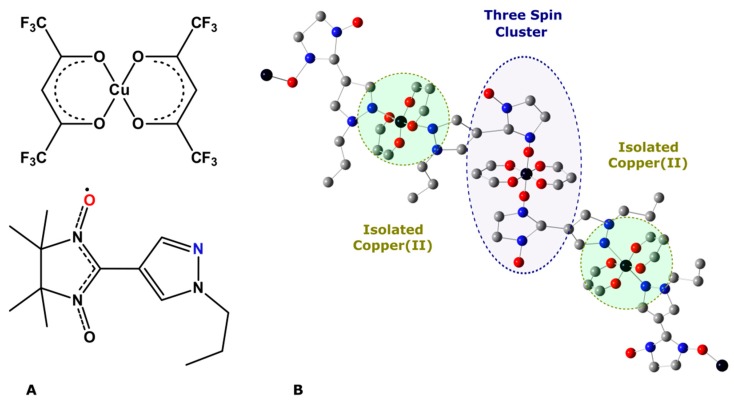
(**A**) Chemical structure of Cu(hfac)_2_ and L^Pr^ units of complex **I**, (**B**) “head-to-head” polymer chain motif of complex **I**, formed by coordination of the L^Pr^ fragment via the nitrogen atom marked in blue (see A) and by coordination of the oxygen atom marked in red (see A) to the second Cu(hfac)_2_ unit. Two types of paramagnetic units can be marked in complex **I**: magnetically isolated copper(II) ion and nitroxide-copper(II)-nitroxide exchange-coupled cluster. Trifluoromethyl, methyl groups and hydrogen atoms are omitted for clarity. Color scheme: black–Cu, blue–N, red–O, gray–C.

For TR EPR experiments we used ~0.5 mm^3^ single crystal of copper(II)-based polymer chain complex Cu(hfac)_2_L^Pr^ (**I**) where Cu(hfac)_2_ is copper(II) bis-hexafluoroacetylacetonate and L^Pr^ is pyrazolyl-substituted nitronyl nitroxide radical. The chemical structure of **I** is shown in Figure 5. Detailed information concerning synthesis procedure, EPR, magnetic, structural, and optical properties of **I** can be found elsewhere [9,10,11,12]. In TR EPR experiments, complex **I** was irradiated by THz macropulses of different length, the THz radiation wavenumber was 76.7 cm^−1^. The absorbance of the sample **I** at this wavenumber was not measured due to the small size of the crystal used. Taking into account that there are no intense absorption bands, but only weak ones in this spectral range [12], complex **I** was assumed to be semi-transparent at the THz radiation used. Therefore, one would expect homogeneous heating of the sample **I** upon THz radiation. The THz waveguide used in experiments collimates THz light onto the sample and simultaneously avoids absorption of THz radiation by the waveguide itself and by any internal (“EPR-sensitive”) parts of the EPR resonator. Thus, the sample is assumed to be the only object in the EPR resonator effectively absorbing THz radiation. Temperature of the sample **I** was 7 K unless otherwise stated.

## 3. Results and Discussion

### 3.1. Generation of Macropulses at Novosibirsk Free Electron Laser (NovoFEL)

**Figure 6:**
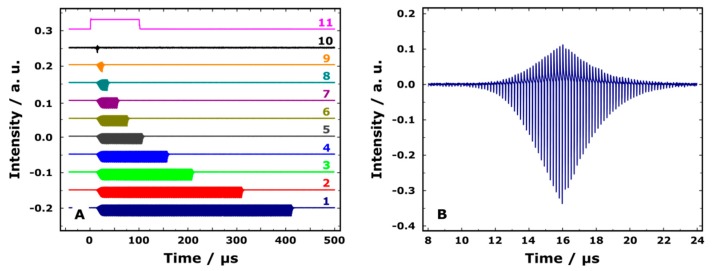
(**A**) Macropulses of THz radiation with wavenumber of 76.7 cm^−1^. Macropulse durations are (1) 400 µs, (2) 300 µs, (3) 200 µs, (4) 150 µs, (5) 100 µs, (6) 70 µs, (7) 50 µs, (8) 30 µs, (9) 20 µs, (10) 10 µs (multiplied by 10 in intensity), (11) trigger signal. Each subsequent pulse is vertically shifted. (**B**) Macropulse with 10 µs duration. The individual pulses of THz radiation with the repetition frequency of 5.6 MHz are clearly visible.

In order to demonstrate the possibilities of an electronic modulation system (EMS), we recorded the THz macropulses with different pulse length at all three FELs of NovoFEL, which correspond to different energy (wavelength) ranges of available radiation. Figure 6A shows macropulses of THz radiation with different length in the range of 10 µs to 400 µs obtained at 76.7 cm^−1^ wavenumber (1st FEL). Pulses with the duration of more than 400 µs are also available. Similar figures for two other FELs are given in the Supporting Information (Figures S3 and S6).

Figure 6B and Figures S4 and S7 in the Supporting Information show the rising and falling edges of the shortest obtained macropulse for the three FELs available. The time resolution of the detector used at the first FEL (Figure 6B) is enough to see the fine structure of the THz macropulse, which consists of a series of short individual pulses at the electron beam frequency. From the analysis of THz macropulse edges, it is possible to obtain the characteristic rise (build-up of generation, the sign of the signal depends on the detector used) and decay (suppressing of generation) times. These two times are linked to the gain and total losses per round-trip of THz wave in the optical resonator, respectively. Calculations of these two parameters were done by the standard procedure described elsewhere [13,14]. Obtained parameters for all three FELs are given in Table 1. Characteristic exponential times and the treatment of the rising and falling edges of THz macropulses are given in Figures S10–S14 of the Supporting Information.

Obtained values reflect the conditions at the time of the experiment, in which the gain of each of the three FELs is certainly not the maximum, and the optical resonator may be slightly misaligned. Nevertheless, the values of the gain and losses given in Table 1 do not contradict to the special measurements of the NovoFEL parameters made earlier [13,14].

### 3.2. Possible Applications of Electronic Modulation of THz Radiation at NovoFEL

The electronic modulation system at NovoFEL provides unique possibilities for controlling and tuning the average power of THz radiation and the repetition rate of THz macropulses directly at the user stations. The possible applications of EMS, used in experiments with THz radiation, include the following options: (i) controlling of the average THz power over the long time period, (ii) formation of short macropulses with highest peak power available, (iii) periodic switching on and off of the THz radiation in the experiments utilizing lock-in detection schemes.

#### 3.2.1. Control of the Average THz Power in CW Experiments

In order to precisely measure the influence of CW THz radiation on different objects, including biological ones [15], it is crucial to have a stable average THz power over a long period of time (hours). However, this is quite a challenging task. Indeed, the generation of THz radiation is determined by fine tuning of a number of different parameters that are sensitive to the long-term stability of the electron beam, heating of optical resonators and other parts, etc. In turn, using EMS, the desired power can be achieved by measuring the average power in real time and then automatically adjusting the number of electron beam pulses with shifted frequency that suppress generation of radiation. For optimal performance, the working average power should be approximately two times lower than the peak power available at the moment in order to have an amplification reserve. Such reserve allows for decreasing the number of shifted electron beam pulses and keeping the same power during а possible NovoFEL detuning over the day.

#### 3.2.2. Formation of Short High-Power THz Macropulses

In addition to studying the cumulative effects of THz on various objects, EMS allows one to routinely perform time-resolved experiments with a time resolution determined by the shortest available THz macropulse. As one can see in Figure 6, 10 µs THz pulses are reachable with typical amplification coefficients of the optical resonators of NovoFEL. Apart from resolution in time, the use of macropulses decreases the average THz power of NovoFEL but keeps its peak value. Such a control of radiation power is important in practically every experiment to prevent overheating of the sample under study or even its irreversible damage. It should also be noted that the impact of individual THz pulses on the sample in the microsecond macropulse can also be investigated, which further extends the temporary resolution of the experiment.

#### 3.2.3. Lock-in Detection

EMS can be used in experiments utilizing lock-in detection schemes where the modulation of THz power at certain frequency is required. The repetition rate of THz macropulses, which is set by the frequency generator directly at the user stations, can be used for lock-in detection of the modulated signal. Since NovoFEL can be operated in CW mode, there is no low frequency limit for macropulse repetition rate. The upper limit is mainly determined by the rise and decay times of the FEL optical resonator and corresponds to 10–50 kHz. The advantage of using a lock-in detection scheme offered by EMS is a possible increase in the signal-to-noise ratio of measured values.

### 3.3. Studying the Spin Dynamics Induced by High-Power THz Macropulses of ~30 µs Duration

NovoFEL is equipped with a number of user stations representing different experimental techniques [16]. Among them, the EPR spectroscopy station allows for the detection of unpaired electrons in various materials and thus can be employed to study the influence of THz radiation onto their spin dynamics [3]. Over the past decades, a number of EPR techniques have been developed and widely used in physics, chemistry, and biology [16,17,18,19,20,21]. The NovoFEL EPR spectroscopy station is capable for so-called steady-state EPR and time-resolved (TR) continuous wave EPR experiments. The TR EPR method is sensitive to transient processes and is able to record a change in MW absorption caused by an external influence. This method is often used in the elucidation of structures and spin dynamics of photoexcited paramagnetic species [19,21]. In most cases, the latter represents short-lived triplet states and radical pairs generated by the laser pulse of the ultraviolet–visible (UV-Vis) range (targeted processes are related to photochemistry). At the same time, THz-radiation of NovoFEL has much less energy quanta and is used to excite either vibrational bands of studied compounds [22] or high-energy EPR transitions [3] in high-spin systems with large zero-field splitting (targeted processes are related to spin system excitation) [23]. Regardless of the type of targeted processes, the absorption of THz light by the sample leads to its heating. It was shown earlier that pulse heating of the sample at helium temperatures is accompanied by strong T-jump TR EPR signals [3]. Typical thermal relaxation times of the EPR sample were shown to be in the range of 2–5 ms at 4–5 K and can reach 20 ms and more at higher temperatures [3]. Disentangling of these indirect thermal effects and the targeted processes (e.g., resonant high-spin system excitation by THz light) requires THz pulses of much shorter duration compared to the typical thermal relaxation times of the sample. The use of very short THz pulses (tens of µs and less) also significantly reduces the overall heat load of the sample and thus allows for utilizing the maximum THz peak power available. Herein, we demonstrate T-jump TR EPR signals induced by THz pulse tens of µs length and compare them with the signals measured earlier using THz pulses with an order of magnitude longer duration.

**Figure 7:**
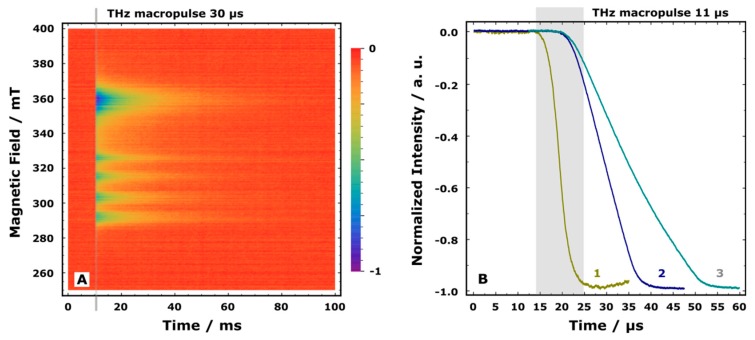
(**A**) Normalized time-resolved electron paramagnetic resonance (TR EPR) spectrum of **I** measured at 7 K using THz macropulses of 30 µs length and 76.7 cm^−1^ wavenumber. Microwave (MW) frequency is 9.79 GHz, MW power is 2 µW, repetition rate of THz macropulses is 5 Hz. (**B**) The initial parts of normalized TR EPR kinetics of **I** at 360 mT obtained using different length of THz macropulses: (1) 11 µs, (2) 20 µs, (3) 30 µs. A strong negative signal is induced by THz radiation, its build-up time reflects the macropulse length. A gray rectangle schematically shows the time range of the negative signal formation for the shortest macropulse used. Experimental parameters are the same as in A.

As was mentioned above, in standard TR EPR experiments, a change in the MW absorption caused by an external influence, such as UV laser pulse (THz pulse in our case) is recorded. The sign and shape of the resulting TR EPR spectrum reflect the difference in the EPR signals of the spin system prior to and after an external stimulus. THz radiation can be effectively absorbed by the vibrational bands of the sample which results in its heating [3]. Heating of the sample gives rise to a change in the population of spin levels that manifests itself as a strong negative signal in the TR EPR spectrum [3]. It should be noted that the population of spin levels equilibrates with a characteristic spin relaxation time. The spin relaxation time in the system under the study is shorter than the length of the THz macropulse. In that case, the TR EPR signal reflects sample temperature changes induced by the latter. Fast spin relaxation is typical for magneto-concentrated compounds such as **I**, which was indirectly confirmed for **I** by failing to observe electron spin echo in pulse EPR experiments at helium temperatures. Figure 7A shows the normalized TR EPR spectrum of **I** measured under THz pulse radiation with a length of 30 µs created by EMS. In this figure, the time axis shows temporal behavior of the signal, which is determined by heating of the sample by THz pulse (indicated by a gray rectangle) and subsequent thermal relaxation process. Thermal relaxation is close to an exponential function with 15 ms characteristic time (Figure S15C of the Supporting Information), which is in agreement with the results obtained earlier [3]. The position of negative features at the magnetic field axis corresponds to CW EPR spectrum of **I** (see Figure S16D of the Supporting Information). At temperatures below 90 K, the CW EPR spectrum is determined by two independent spin systems (Figure 5), namely magnetically-isolated copper(II) ion with the resolved hyperfine structure (magnetic field region of 280–340 mT) and exchange-coupled nitroxide-copper(II)-nitroxide cluster (magnetic field region of 340–380 mT) [3]. Magnetic field and time cross-sections obtained from Figure 7A are given in Figure S15 of the Supporting Information.

The initial part of kinetics, which corresponds to the build-up of negative TR signal, allows us to characterize the THz macropulse length directly in the TR EPR experiment. Figure 7B shows the initial parts of three different normalized TR EPR signals of **I** obtained at 360 mT. Each of them reflects the macropulse length used at the moment of the experiment, because changing of the sample temperature at such timescales occurs only due to direct absorption of THz radiation. As it can be seen from Figure 7B, macropulses with lengths as short as 10 µs can be routinely available using EMS. It is also interesting to note that the starting points of the negative TR EPR signal are different for the 11 µs pulse and two other pulses. The reason for this is the different amplification coefficient of NovoFEL in these experiments since shorter THz macropulses require a higher amplification coefficient.

**Figure 8:**
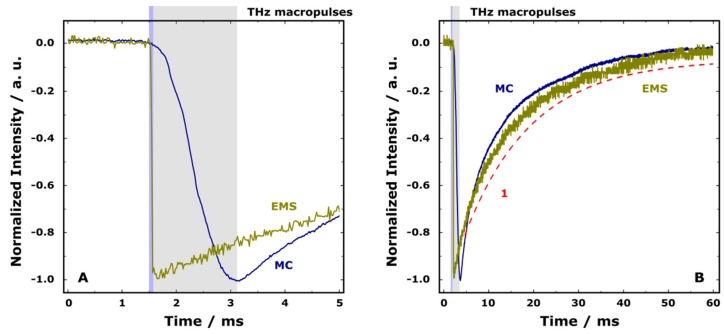
(**A**) Comparison of the initial parts of normalized TR EPR kinetics of **I** at 360 mT obtained by the electronic modulation system (EMS) and mechanical optical chopper (MC, see reference [3] for details) using typical pulse lengths for these two approaches of radiation modulation. Blue and gray rectangles schematically show THz macropulse lengths for EMS and MC, respectively. Experimental parameters are given in the Figure 7 caption. (**B**) The same as A at different timescale. Curve (1) shows one-exponential fit (vertically shifted) of the TR EPR kinetics obtained by EMS. The characteristic time is 15 ms.

Finally, based on the TR EPR signal of **I**, it is possible to directly compare the typical pulse lengths of THz radiation obtained by EMS and optical mechanical chopper (MC) with fixed duty cycle and variable rotation frequency. A modulation system based on mechanical chopping of radiation was previously implemented at the TR EPR station [3]. Figure 8A shows the comparison of the initial parts of TR EPR signals of **I** obtained at 360 mT using EMS and MC. Further development of thermal relaxation is shown in Figure 8B. One can see that TR EPR kinetics obtained with a short THz macropulse (EMS) can be reasonably simulated with one-exponential fit. In turn, when a longer THz macropulse is used (MC), TR EPR kinetics shows more complicated character. Indeed, while the kinetics “tail” has a similar characteristic time as for EMS (~15 ms), the initial part of the decay develops noticeably faster. It can be caused by a significant change in the sample temperature, which results in the subsequent complex (multi-exponential) manner of the thermal relaxation of the sample.

As was already mentioned, the routinely available pulse length obtained by EMS is about 10–30 µs. At the same time, the shortest reasonable macropulse reachable by MC is about 300–400 µs, being determined by typical rotation frequency. The pulse length and repetition rate in the electronic modulation regime are two independent parameters, which makes it possible to use short pulses with low repetition rate and vice versa. The use of short THz macropulses prevents the sample from overheating even when the maximum peak THz power is utilized. One more advantage of EMS compared to MC is better time resolution in TR EPR experiments, achieved due to more than 10 times shorter THz macropulse.

## 4. Conclusions

In this work, we developed and implemented the approach allowing one to create THz macropulses at NovoFEL with any repetition rate and almost any individual pulse length. The proposed electronic modulation system is based on a periodic shift of the phase of the electron bunch injection. Such a shift suppresses lasing and forms macropulses from quasi-continuous radiation of NovoFEL. The system is directly embedded into the electronic infrastructure of NovoFEL and can be triggered directly on user stations. For experimentalists, such an electronic modulation system provides a unique possibility, e.g., to control the average power of THz radiation over a long period of time and to create macropulses of a duration as short as 10 µs.

In order to characterize EMS, a series of macropulses with different durations from 10 to 400 µs were measured for three available frequency ranges. Using the rising and falling edges of typical macropulses, the calculations of the gain and total losses were done for three optical resonators. Obtained characteristics agree with the data obtained earlier.

The use of short macropulses was exemplified for TR EPR spectroscopy. The heating of copper(II) complex by the absorbed THz radiation and its subsequent thermal relaxation were measured. These data were also compared with the results of another modulation system based on mechanical optical chopper that was implemented previously. We have demonstrated that the new electronic modulation system allows reproducible microsecond THz macropulses to be routinely applied at user stations of NovoFEL. This gives new opportunities to perform different experiments with improved time resolution and stability of THz radiation.

## Figures and Tables

**Table 1:** The gain and total losses per round-trip of THz wave for three FELs of the NovoFEL facility.

FEL	Gain/%	Losses/%
1st	20.0	10.3
2nd	8.9	4.1
3rd	19.6	9.7

## Supplementary Materials

The following are available online at https://www.mdpi.com/1996-1944/12/19/3063/s1, Figure S1: Photograph of EPR spectroscopy station at NovoFEL, Figure S2: Spectrum of radiation used in the experiments at the first FEL, Figure S3: Macropulses of THz radiation with wavenumber of 239 cm^−1^, Figure S4: The rising and falling edges of THz macropulse from the second FEL, Figure S5: Spectrum of radiation used in the experiments at the second FEL, Figure S6: Macropulses of THz radiation with wavenumber of 1125 cm^−1^, Figure S7: The rising and falling edges of THz macropulse from the third FEL, Figure S8: Spectrum of radiation used in the experiments at the third FEL, Figure S9: Calculation of losses for the first FEL, Figure S10: Gain calculation for the first FEL, Figure S11: Calculation of losses for the second FEL, Figure S12: Gain calculation for the second FEL, Figure S13: Calculation of losses for the third FEL, Figure S14: Gain calculation for the third FEL, Figure S15: Time and magnetic field cross-sections of TR EPR signal of complex **I**, Figure S16: CW EPR spectrum of complex **I**.
