# Supplementary material for: Electronic Modulation of THz Radiation at NovoFEL: Technical Aspects and Possible Applications
Source: Materials (Basel). 2019 Sep 20;12(19):3063. doi: 10.3390/ma12193063 (PMC6803909; doi:10.3390/ma12193063)
Supplement: Supplementary file 1 [file materials-12-03063-s001.pdf]

materials

Supplementary

Electronic Modulation of THz Radiation at NovoFEL: Technical Aspects and Possible Applications

Oleg A. Shevchenko [1,*], Anatoly R. Melnikov [2,3,4], Sergey V. Tararyshkin [1], Yaroslav V. Getmanov [1,4], Stanislav S. Serednyakov [1], Evgeny V. Bykov [1], Vitaly V. Kubarev [1,4], Matvey V. Fedin [2,4] and Sergey L. Veber [2,4,*]

[1] Budker Institute of Nuclear Physics SB RAS, 630090, Novosibirsk, 9, Acad. Lavrentieva Ave., Russia; shevchen@mail.ru (O.A.S.); s.v.tararyshkin@inp.nsk.su (S.V.T.); y_getmanov@mail.ru (Y.V.G.); S.S.Serednyakov@inp.nsk.su (S.S.S.); e.v.bykov@inp.nsk.su (E.V.B.); vitaly.kubarev@yandex.ru (V.V.K.);
[2] International Tomography Center SB RAS, 630090, Novosibirsk, 3a, Institutskaya Str., Russia; anatoly.melnikov@tomo.nsc.ru (A.R.M.); mfedin@tomo.nsc.ru (M.V.F.); sergey.veber@tomo.nsc.ru (S.L.V.);
[3] Voevodsky Institute of Chemical Kinetics and Combustion SB RAS, 630090, Novosibirsk, 3, Institutskaya Str., Russia; melnikov@kinetics.nsc.ru
[4] Novosibirsk State University, 630090, Novosibirsk, 1, Pirogova Str., Russia
* Correspondence: shevchen@mail.ru (O.A.S.); sergey.veber@tomo.nsc.ru (S.L.V.); Tel: +7-383-333-1460

Contents

A. Photograph of the experimental setup S2
B. First FEL S3
C. Second FEL S4
D. Third FEL S5
E. Gain and total losses for all three FELs S6
1. First FEL S6
2. Second FEL S7
3. Third FEL S8
F. Additional experimental figures S9

A. Photo of the Experimental Setup

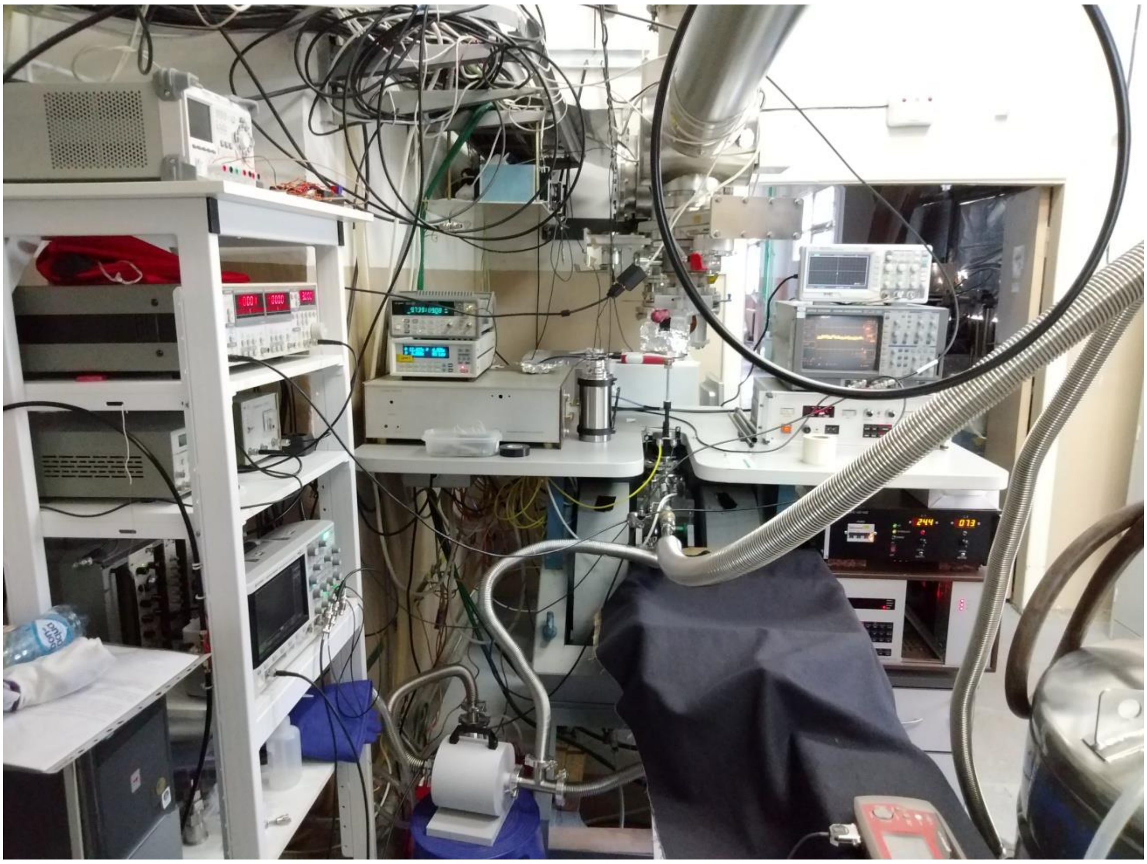

Figure S1. Photograph of the EPR spectroscopy station at NovoFEL.

B. First FEL

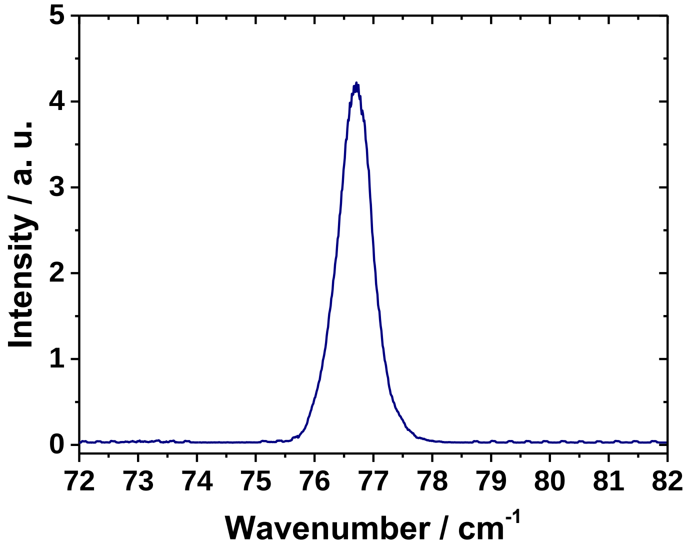

Figure S2. Spectrum of radiation used in the experiments at the first FEL of the NovoFEL facility.

C. Second FEL

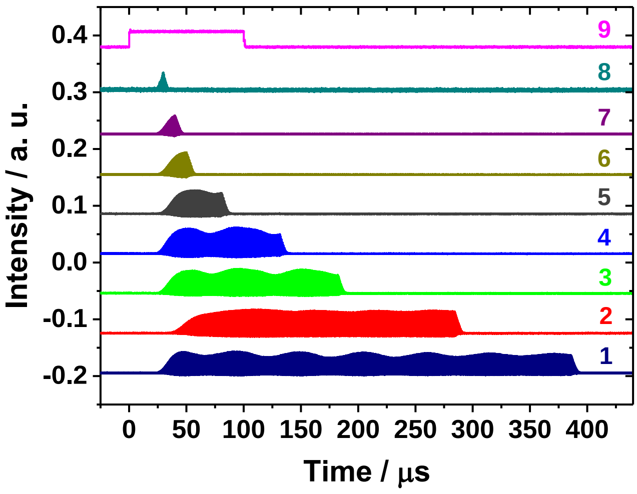

Figure S3. Macropulses of THz radiation with wavenumber of 239 cm^{-1}. Pulse durations are (1) 400 µs; (2) 300 µs; (3) 200 µs; (4) 150 µs; (5) 100 µs; (6) 70 µs; (7) 60 µs; (8) 50 µs (multipled by 5); (9) trigger signal. Each subsequent pulse is vertically shifted.

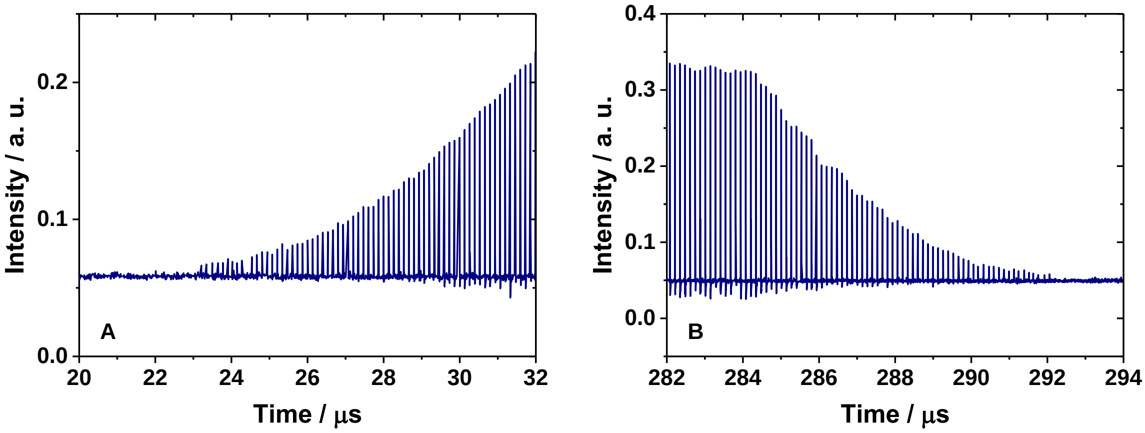

Figure S4. (**A**) The rising edge of the macropulse with 300 µs duration; (**B**) The falling edge of another macropulse with 300 µs duration. The individual pulses of THz radiation with the frequency of 7.5 MHz are clearly visible.

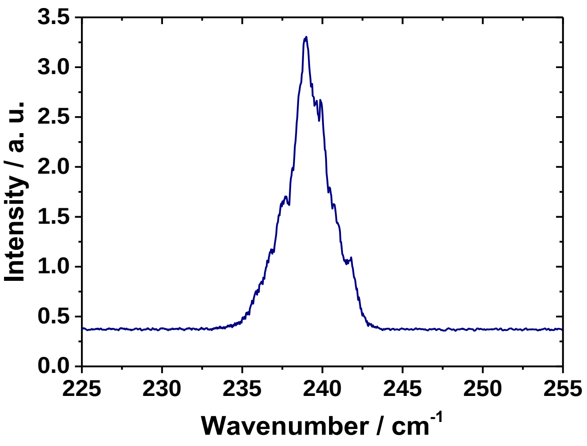

Figure S5. Spectrum of radiation used in the experiments at the second FEL of the NovoFEL facility.

D. Third FEL

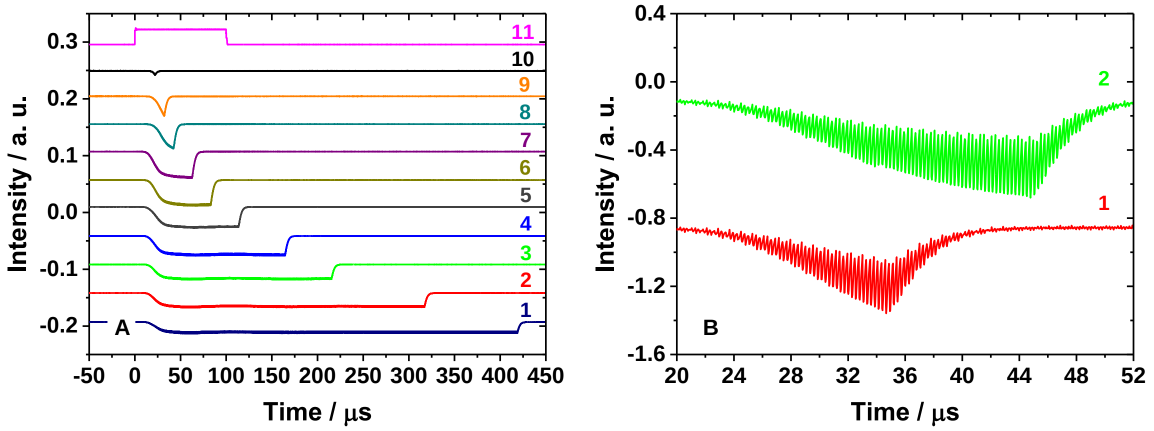

Figure S6. (**A**) Macropulses (envelop) of THz radiation with wavenumber of 1125 cm^{-1}. Pulse durations are (1) 400 µs; (2) 300 µs; (3) 200 µs; (4) 150 µs; (5) 100 µs; (6) 70 µs; (7) 50 µs; (8) 30 µs; (9) 20 µs; (10) 10 µs (multipled by 10); (11) trigger signal. Each subsequent pulse is vertically shifted. No pick-up coil synchronization was used; (**B**) The rising and falling edges of the macropulses with (1) 20 µs and (2) 30 µs duration. The individual pulses of THz radiation with the frequency of 3.8 MHz are clearly visible.

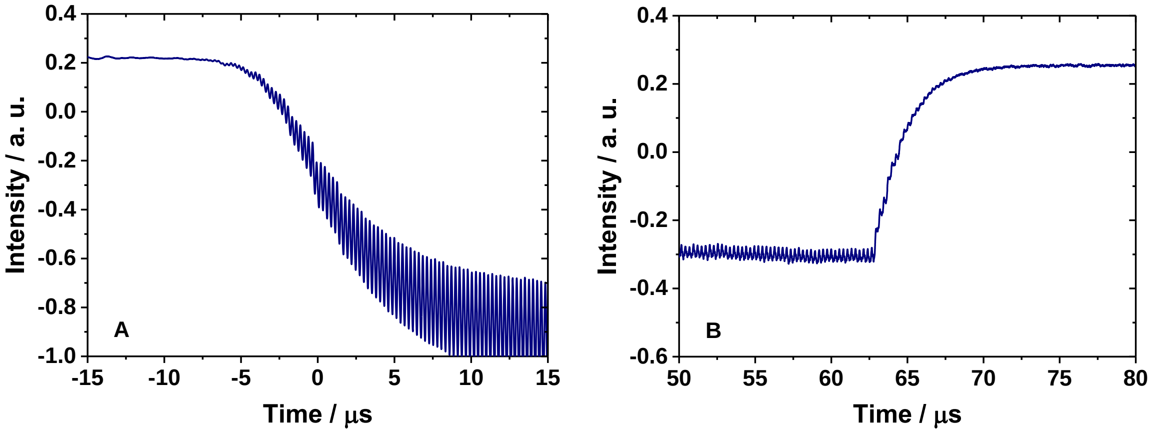

Figure S7. (**A**) The rising edge of the macropulse with 100 µs duration; (**B**) The falling edge of another macropulse with 100 µs duration. No pick-up coil synchronization was used for **B**.

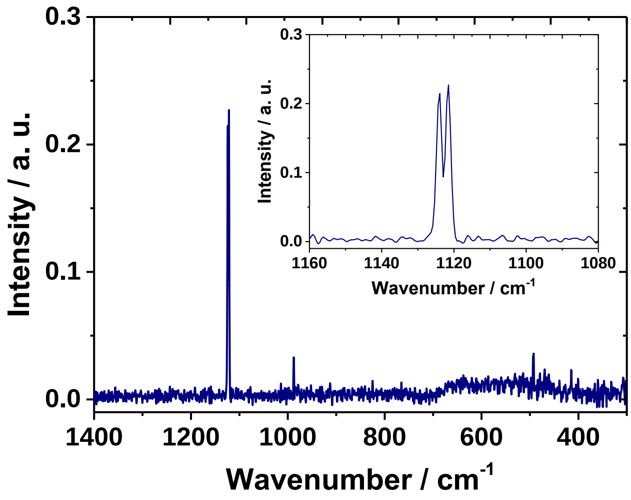

Figure S8. Spectrum of radiation used in the experiments at the third FEL of the NovoFEL facility. Inset shows closer view of the spectrum.

E. Gain and Total Losses for all Three FELs

1. First FEL

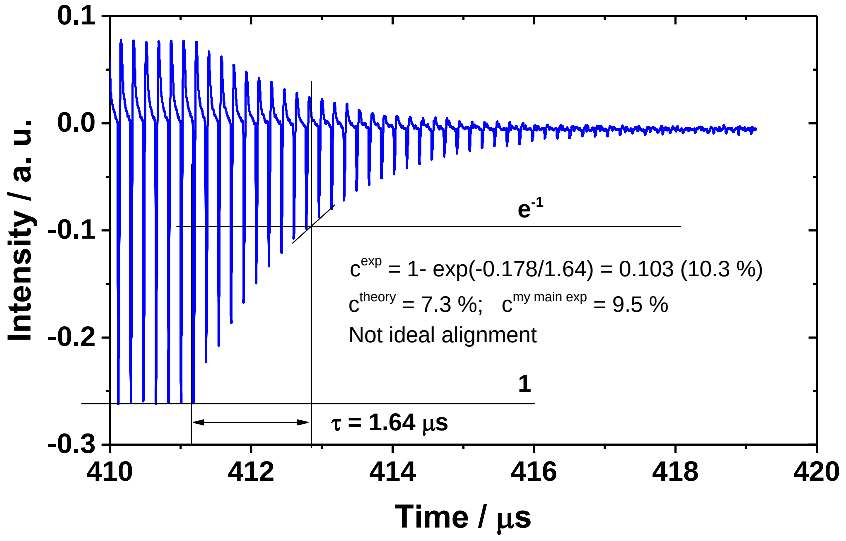

Figure S9. Calculation of losses for the first FEL. Characteristic exponential time is 1.64 μs.

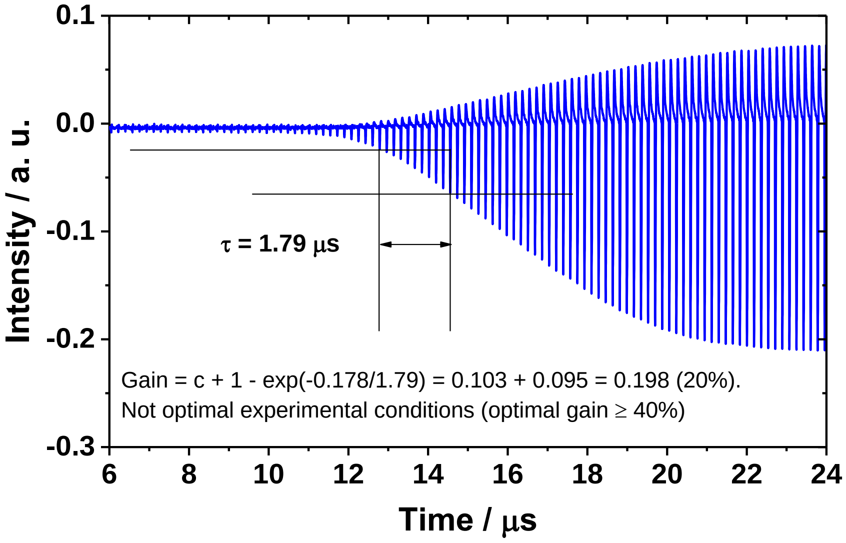

Figure S10. Gain calculation for the first FEL. Characteristic exponential time is 1.79 μs.

2. Second FEL

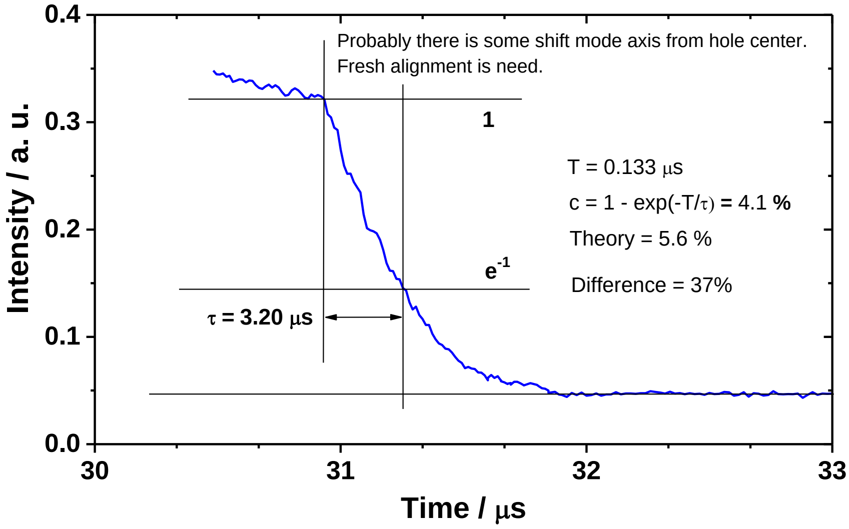

Figure S11. Calculation of losses for the second FEL. The signal envelope was obtained by FindPeaks function of Wolfram Mathematica 10. Characteristic exponential time is 3.20 μs.

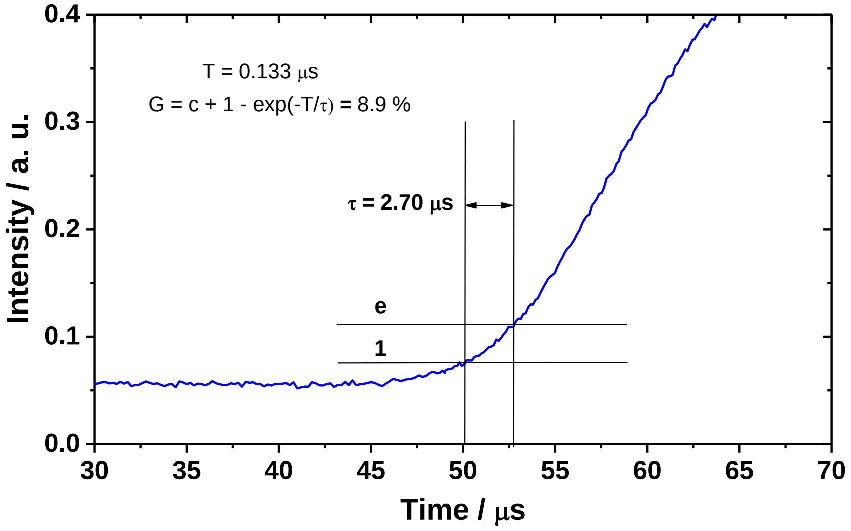

Figure S12. Gain calculation for the second FEL. The signal envelope was obtained by FindPeaks function of Wolfram Mathematica 10. Characteristic exponential time is 2.70 μs.

3. Third FEL

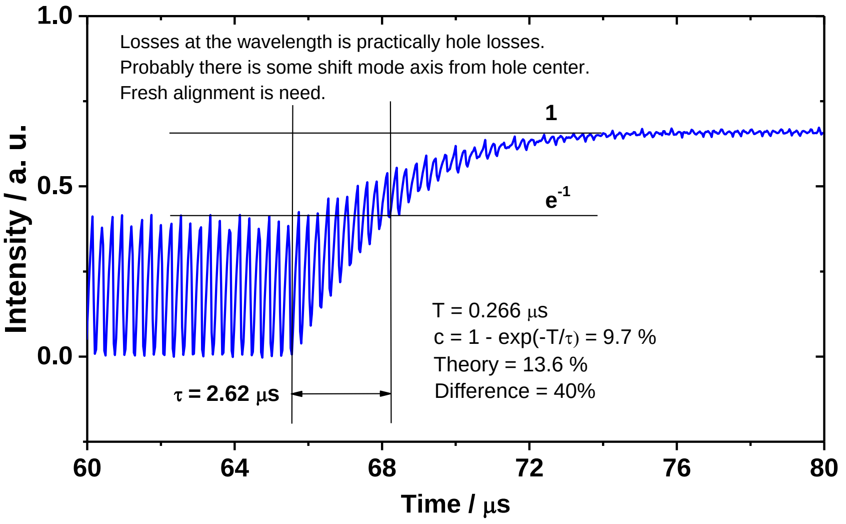

Figure S13. Calculation of losses for the third FEL. Characteristic exponential time is 2.62 μs.

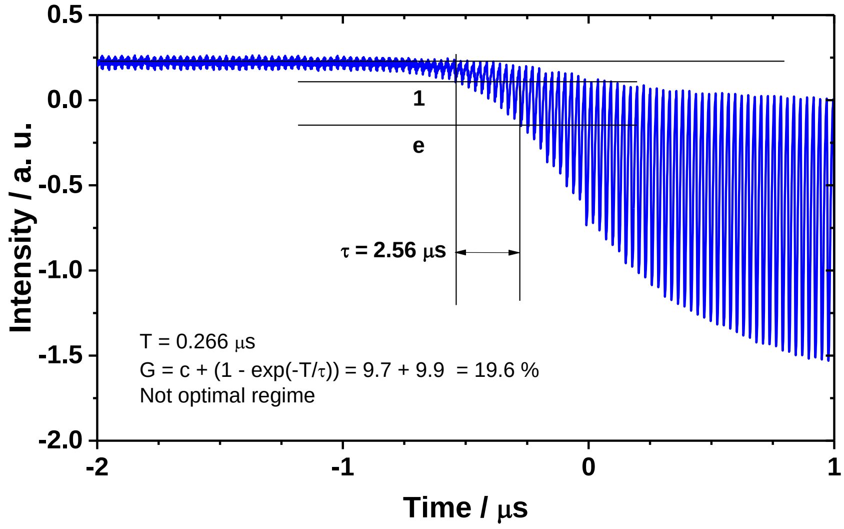

Figure S14. Gain calculation for the third FEL. Characteristic exponential time is 2.56 μs.

F. Additional Experimental Figures.

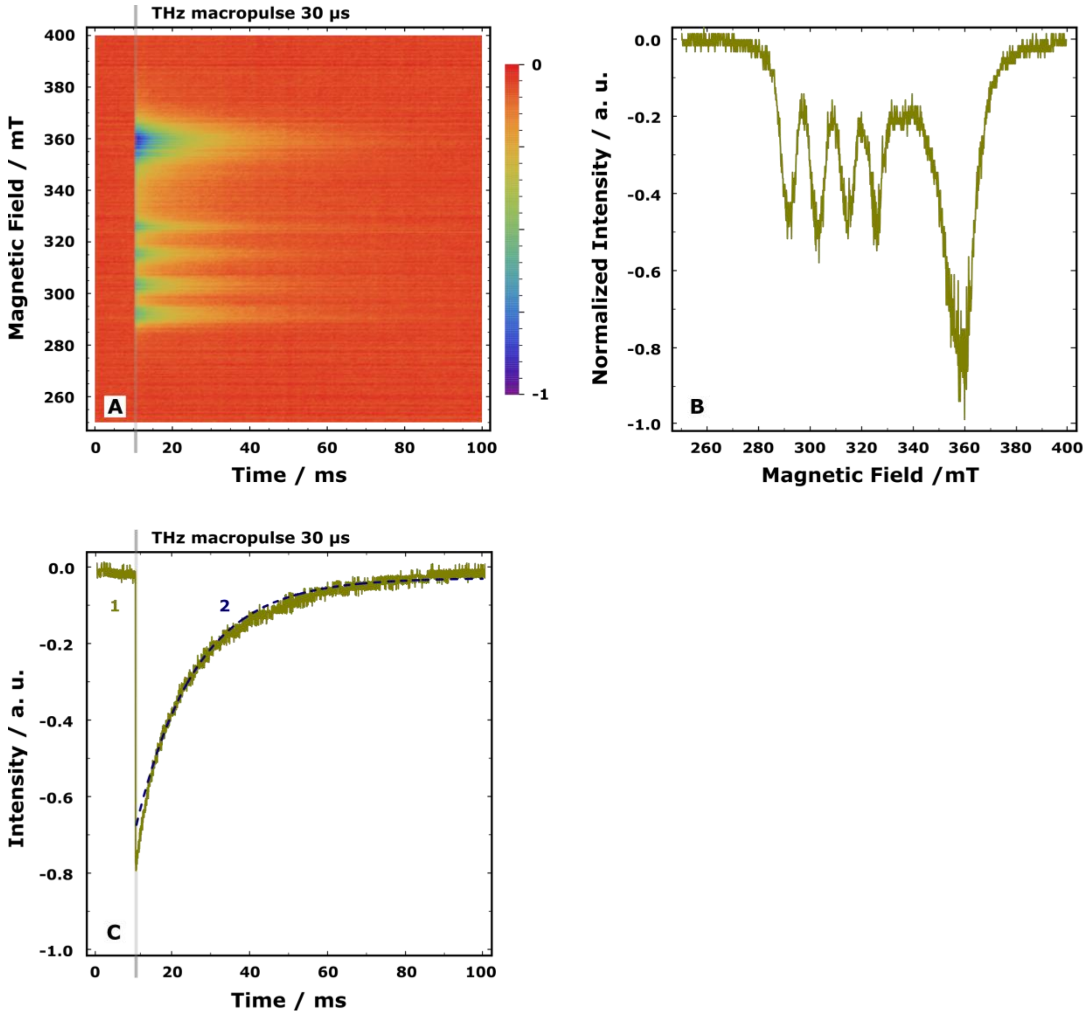

Figure S15. (**A**) Normalized TR EPR spectrum of Cu(hfac)$_2$L^{Pr} (see Figures 5 and S16 for the structure) measured at 7 K using THz macropulses of 30 μs length and 76.7 cm^{-1} wavenumber. MW frequency is 9.79 GHz, MW power is 2 μW, repetition rate of THz macropulses is 5 Hz; (**B**) Magnetic field cross-

section of **A** at the time of the maximal signal; (**C**) Time cross-section of **A** at 360 mT (1) and one-exponential fit with the characteristic time 15 ms (2).

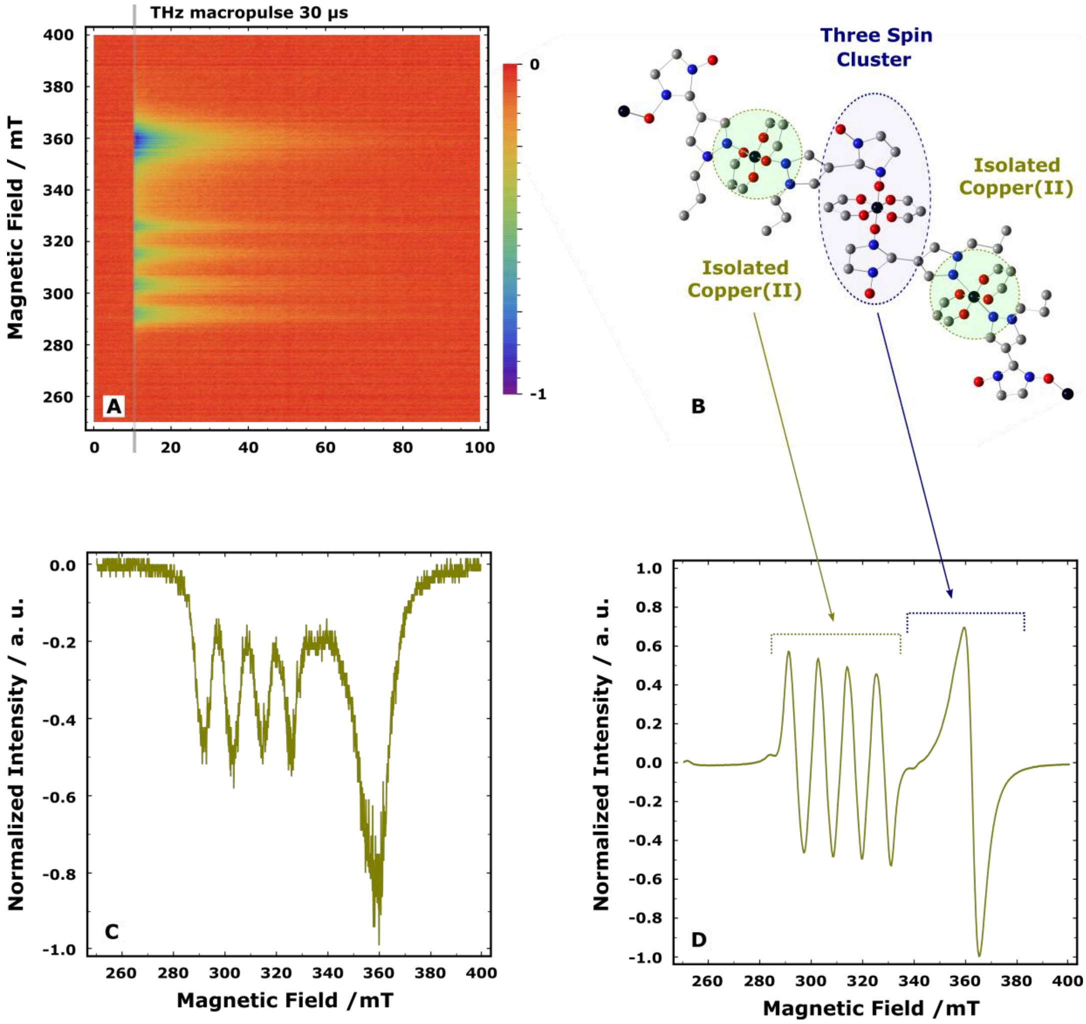

Figure S16. (**A**) Normalized TR EPR spectrum of Cu(hfac)$_2$L^{Pr} measured at 7 K using THz macropulses of 30 μs length and 76.7 cm^{-1} wavenumber. MW frequency is 9.79 GHz, MW power is 2 μW, repetition rate of THz macropulses is 5 Hz; (**B**) Chemical structure of Cu(hfac)$_2$L^{Pr} with the paramagnetic centers circled: magnetically isolated copper(II) ion and exchange-coupled nitroxide-copper(II)-nitroxide spin triad (see Figure 5 of the main text for details); (**C**) Magnetic field cross-section of **A** at the time of the maximal signal; (**D**) Normalized continuous wave EPR spectrum of Cu(hfac)$_2$L^{Pr}. Temperature is 7 K, MW frequency is 9.79 GHz, MW power is 2 μW, modulation amplitude is 0.2 mT, modulation frequency is 100 kHz, no THz radiation was applied.
